# The Diagnosis of Peptic Ulcer and Its Bearings on Treatment

**Published:** 1923-03

**Authors:** T. Carwardine

**Affiliations:** Surgeon to the Bristol Royal Infirmary


*"Scire est nescire, nisi id me*
*Scire alius sciret.*

MARCH, 1923.

### THE DIAGNOSIS OF PEPTIC ULCER AND ITS BEARINGS ON TREATMENT.

BY

T. CARWARDINE, M.S.,

*Surgeon to the Bristol Royal Infirmary.*

PAIN, hæmatemesis and vomiting were regarded formerly as signs of peptic ulcer. They are known now to be utterly unreliable as signs, both singly and collectively.

The symptomatology of peptic ulcers needs to be written afresh on a clean slate, and more consideration paid to various conditions which mimic the symptoms. On short reflection it is obvious that there is something radically wrong. Duodenal ulcer is far more common than gastric ulcer, and chronic gastric ulcer is rather more common in men than in women, yet how much more frequently are women treated than men for the condition when it has not been proved to exist! As Carman says, no other organ in the human body to-day has been accused of so many disorders which it never had as the stomach.[1] This applies particularly to women, with more numerous reflex possibilities. There is a difference between the sex incidence of peptic ulcer diagnosed clinically and that determined by actual inspection at operation or post-mortem.

During the last year, of the cases referred to me for operation for supposed peptic ulcer (commonly diagnosed gastric), in only about one-third has the diagnosis proved correct, and in eight consecutive cases only two showed the lesion diagnosed and for which the patients had been treated, maybe for years.

When really present peptic ulcer is a serious disease. It is probably true that if cure does not take place in four or five weeks under medical treatment 25 per cent. of uncured cases will die unless surgical aid is resorted to. Of the 500 cases at the London Hospital in five years (1897–1902) 18 per cent. died and 40 per cent. relapsed.[3] 10 per cent. of the deaths were due to perforation, and of the relapsing cases some must have ended in cancer.

The signs of peptic ulcer may be classified as on opposite page, and must include the X-ray findings which have been grouped clearly by Carman.[2]

In this classification the following points may be noted :—

1. The **direct** signs are either X-ray findings or complications.

2. The **indirect** signs are reflex, inferences from X-rays, and effects of involvement of neighbouring organs.

3. The **corroborative** are clinical (and chemical) and X-ray signs, chiefly of retention.

4. The **groups** are classified: *horizontally*, for comparison of gastric and duodenal signs; and *vertically* in alignment, for the signs and symptoms in the first column and for X-ray findings in the second column.

**X-ray Diagnosis.**—X-ray photography is becoming by far the most accurate means of diagnosis, and is destined

### SIGNS OF PEPTIC ULCER.

### GASTRIC. DUODENAL.

**ACUTE.**

Young Women, 15–25, *e.g.* Burns.

**CHRONIC.**

### GASTRIC.

**A. DIRECT.**

1. NIL.
2. COMPLICATIONS.
   Hemorrhage.
   Perforation.
   Stenosis and Dilatation.
   Tumour.
   Vomiting (Retentive).
3. X-RAY. Pathognomonic.
   1. NOTCH.
   2. NICHE.
   3. ACCESSORY POCKET.

**B. INDIRECT.**

1. NIL.
2. REFLEX.
   Hyperalgesia *to pinch.*
   Hyperæsthesia and Tenderness.
   Pain (? + *increased tension*).
   Rigidity (*reflex*).
   Vomiting (*reflex*).
3. INVOLVEMENT.
   Pancreas
   Subphrenic Abscess.
4. X-RAY. Diagnostic.
   1. ORGANIC HOUR-GLASS.
   2. SPASMODIC HOUR-GLASS. *Intrinsic (Belladonna Test).*
   3. GASTROSPASM.

**C. CORROBORATIVE.**

1. NIL.
2. CLINICAL.
   Succussion Splash.
   Retention, 6-hour +
   Visible Peristalsis.
   Hyperacidity (*exceptional*).
   Test-Meal (*little value*).
   Wasted + Anæmic.
3. X-RAY. Not diagnostic.
   1. RETENTION, 6-HOUR +
   2. HYPOTONUS.
   3. IRREGULAR PERISTALSIS
   4. STOMACH SHORT, WIDE, BAGGY.

### DUODENAL.

**A. DIRECT.**

1. NIL.

2. COMPLICATIONS.

   Hemorrhage, Perforation } Chief risks.

   Tenderness. Sometimes, slight.

   Stenosis, Tumour } Rare.

3. X-RAY. Pathognomonic.

   1. DEFORMITY.

   2. DIVERTICULUM.

**B. INDIRECT.**

1. NIL. *Quiescent intervals.*
2. REFLEX.
   Hyperalgesia *to pinch.*
   Hyperæsthesia and Tenderness.
   Pain. "*Hunger*," "*Nocturnal.*"
   Hyperacidity. *Water brash.* $Na_2CO_3$
   Flatulence. *Vomiting rare.*
3. INVOLVEMENT.
   Gall-bladder. *Cholecystitis.*
   Subphrenic Abscess.
4. X-RAY. Diagnostic.
   GASTRIC HYPER-PERISTALSIS (*3 or more waves*).

**C. CORROBORATIVE.**

1. NIL.
2. CLINICAL.
   Relief by Alkalis.
   Hyperacidity.
   Test-Meal (*undoubted value*).
   May look well, *sometimes fat.*
3. X-RAY. Diagnostic.
   1. RETENTION, 6-HOUR +
   2. RAPID PASSAGE *at first.*
   3. HYPER-PERISTALSIS.
   4. NORMAL GASTRIC OUTLINE
   5. STOMACH LONG, TUBULAR.

**JEJUNAL.**

to exceed in precision all other clinical evidences. It is in comparative infancy as yet, but is going very strongly. Technique has improved, rapid exposures with consequent sharpness of outline are possible, and radiographers are accumulating experience in interpretation. Nevertheless, in the majority of cases the conclusions are based upon *indirect* effects at present, the reading of which requires considerable experience and judgment. It is at least a fascinating pursuit, and in a study of nearly 7,000 cases of dyspepsia during six months at the Mayo Clinic Carman claims to have made both positive and negative diagnosis accurately in 96 per cent. of cases.

**Clinical Signs and their Mimicry.**—We may now take these in order and discuss them critically :—

1. **Reflex symptoms of the disease.**—It is at once apparent that many of the manifestations are of the nature described by Mackenzie. (1) *Viscero-sensory :* pain, hyperalgesia, and their radiation. (2) *Viscero-motor :* rigidity and exaggerated reflexes. (3) *Viscero-central :* vomiting, depression. The reflexes expressed in the somatic sensory nerves have their origin in the three subdivisions of the autonomic nervous system (Langley [4]) : the sympathetic, the parasympathetic (or extended pneumogastric) and the enteric. The sympathetic reflexes concern chiefly the sensibility and musculature of the parietes ; the parasympathetic reflexes concern the vomiting, cardiac effects, headache and scalp tenderness (Head [5]), and in a direct way govern the tonicity and peristalsis of a hollow viscus ; and the enteric reflexes (Langley) are largely associated with the condition of one enteric segment with another (Keith's nodes and myenteric plexus [6]).

2. **Hyperalgesia** (to pinch of uplifted skin).—In describing the hyperalgesic areas of the abdomen, first discovered by Mackenzie in connection with gall-bladder affections, Ligat indicates an area in the mid-epigastrium which may be a vestigial enteric reflex, having vertical extension but not segmental distribution.[7] He states that he found it in 50 out of 80 cases of gastric and duodenal ulcer, that the area indicates the organ at fault, and that the hyperalgesia is a more reliable guide than the history. My observations agree with those of Sherren, that hyperalgesia is no certain guide to gastric ulcer.[8] I am disposed to regard the hyperalgesia as associated with increased tonicity and peristalsis of the gastro-duodenum, even if it be due to an extra-gastric cause such as appendix trouble.

A woman sent in for gastric ulcer, with vomiting and violent pain, had hyperalgesia (and tenderness) in Ligat's epigastric area, and also over the 10th dorsal spine. No ulcer was found, the stomach exhibited spasm, the appendix was removed, both hyperalgesic areas disappeared after the operation, she has been able to eat anything since and remains well.

A patient with adhesions following a former gastro-enterostomy had epigastric hyperalgesia to pinch. The adhesions were set free, and the patient's hyperalgesia and pain disappeared after operation, although there was a thick mass near the pylorus representing the former ulceration.

3. **Muscular Rigidity.**—This Viscero-motor reflex is similar to that which occurs with joint troubles (Hilton's Law[9]), but is only met with in peptic ulcer when the peritoneum is involved. With sudden perforation there is immediate and extreme rigidity, which may pass off until the peritoneum is inflamed.

4. **Tenderness** from cutaneous hyperæsthesia is a Viscero-sensory reflex,[10] and may be present in the epigastrium, or in the back over the 7th, 8th, and 9th ribs, where it corresponds with the posterior primary division of the 6th, 7th and 8th dorsal nerves. But it is *a very unreliable sign* of peptic ulcer, and may be absolutely misleading.

A lady had such extreme tenderness in the left upper epigastrium with vomiting that the diagnosis of gastric ulcer was agreed upon. There was no ulcer, only numerous omental adhesions to an old scar. The patient was well for a month after operation, then the vomiting and symptoms returned.

The difficulty of assigning the reflex phenomena to a particular organ is enhanced in the upper abdomen by the limited area of the dorsal cord involved in the reflex. It seems to be definitely established (for pain) that in the dog the whole of the mesenteric supply of the viscera of the upper abdomen as far as the middle of the small intestine is connected centrally with only two dorsal segments, the 6th and 7th (Kappis[11]). On the theory of the radiation of impulses from one cell to another in so limited an area it is conceivable that several organs may give similar reflexes. An epigastric reflex is not necessarily a gastric one.

5. **Pain.**—An ulcer of the leg is painful because it involves somatic sensory nerves. A peptic ulcer must be essentially painless because a hollow viscus is insensitive to tactile and painful stimuli.

Experimental physicians have taught this (Mackenzie, Head, Crile[12] and others), and Hurst (Goulstonian Lectures[13]) showed that the only adequate cause of true visceral pain is tension ("vital" contraction against tension—Mackenzie). An ulcer of the stomach or bowel may be touched, cauterised, or treated with strong acids without pain. Surgeons remove large portions of extruded bowel painlessly, and open the gut without any sensation whatever, in the absence of anæsthesia, except when the mesentery is dealt with. With peptic ulcer remaining constant the patient experiences exacerbations of pain. In duodenal ulcer the pain is actually relieved when food is passing over the ulcer. Wilkie cured pain of thirty years' duration by an anastomosis which left the existing ulcer alone.[14] In food-allergy a subcutaneous injection of the extract causes abdominal pain.[15] Muller and Ravdin found an absence of previous pain in nearly 25 per cent. of cases of perforated ulcer.[16]

Some years ago a doctor sent his servant under my care with sudden perforation, with no previous pain. A nurse, with no previous pain, died recently from hemorrhage from a gastric ulcer the size of a threepenny-piece.

*True visceral pain* has only been proved to be due to one cause—contractions against tension or resistance (Cannon and Washburn[17]). An extra-visceral and accessory factor is probable—mesenteric drag (Tyrrell Gray[18]). In hyperchlorhydria, hypertonicity and in the pangs of hunger we have the contractions causing the only known effective stimulus for visceral pain, the pneumogastric initiating the stimulus and the painful sensations being conveyed by the sympathetic. The " bowels of compassion " have thus a physiological explanation.

6. **Hemorrhage** (Hæmatemesis and Melæna).—This is a fruitful source of error. Operative experience shows quite definitely that at least 50 per cent. of cases of hæmatemesis and melæna are *not* due to chronic peptic ulcer. In the absence of alcoholism, blood and splenic disease, the appendix and gall-bladder are responsible for most cases of bleeding unassociated with chronic ulcer.

The *Appendix.*—In 5 out of 24 cases of appendix dyspepsia the patients had vomited blood. In one case the stomach was opened and found to be " weeping blood " (Paterson[19]).

A woman, aged 40, seen three years ago, had indigestion for twelve months after every meal at the age of 17, and finally brought up blood. At 19 she was treated for gastric ulcer. At 30 she vomited " 4 pints " of blood. At 31 had severe hæmatemesis and was in bed for six months. At 38 there was recurrence, followed by two years of constant pain to the left of the umbilicus and through to the back, increased directly after food. At the operation over two years ago there were persistent and prolonged contractions of the stomach, which was opened, but no ulcer found. The appendix, adherent and kinked, was removed. Her doctor writes : " She has had no further return of the hæmatemesis, and is able to do more than she was before."

A gentleman, over 70, had indigestion all his life, the pain being relieved by alkalis. Six months ago he had hæmatemesis and melæna. At the consultation there was a chamber-pot a quarter full of blood. A perforated and bleeding duodenal ulcer was diagnosed, but at the operation he had a gangrenous appendix with stinking pus in the pelvis and right loin. No lesion of the duodenum could be felt, and about an hour after he turned pale and died, apparently from recurrent hemorrhage.

A patient sent in with hæmatemesis and the diagnosis of ulcer "sighed and died" the afternoon before the proposed operation. At the post-mortem there was no ulcer. The stomach was hyperæmic, the intestines were purpuric, and both were oozing blood. No emboli or infarcts.

The *Gall-bladder* is responsible for more cases of hæmatemesis than we realise. Rankin had 55 cases of gall-bladder disease with hemorrhage; in 20 the gall-bladder disease was not diagnosed, but the bleeding ceased in most cases after operation.[20]

A history of bleeding may be obtained in 5 per cent. of gall-bladder cases (Crispin[21]), and cases have been recorded by Judd,[22] Deaver,[23] Kelling,[24] and others.

A woman had her appendix removed in London, and later an operation for stomach trouble with bleeding. In the summer of 1922 a colleague asked me to operate on her: she was bleeding, had hyperæsthesia in the *left* hypochondrium, and possible jejunal ulcer was diagnosed. But we found that no gastro-enterostomy had been done. Last December her doctor sent her in again with a note: "She has very definite symptoms of gastric ulcer now and had a severe hemorrhage this morning." But on admission she developed pyrexia with pain and tenderness over the gall-bladder. After waiting some time I removed the gall-bladder, which was thickened, fatty, "strawberry," and buried in congenital adhesions, with some fluid in the peritoneum around. She left on the road to recovery without her gall-bladder, after a diagnosis of peptic ulcer on perhaps three occasions.

In several gall-bladder cases which have been diagnosed as peptic ulcer I have found the gall-bladder adherent by congenital or inflammatory bands in such a way as to obstruct the duodenum. Occasionally the gall-bladder has been buried completely—even for a time indistinguishable from the intestine to which it was adherent. One patient had a seven years' history of supposed duodenal ulcer.

On the other hand, a patient with the diagnosis of gall-stones with pain between the shoulders, has turned out to have a duodenal ulcer. A physician recorded his own symptoms—diagnosed as cholelithiasis amongst other things shortly before his duodenal ulcer perforated.[25]

*The Pancreas* gave rise to symptoms diagnosed as peptic ulcer in the following cases :—

A patient had recurrent *hæmatemesis* for years. No peptic ulcer could be found. There was free fluid in the right hypochondrium. The common duct was dilated with large veins over it, above the part running *through* the head of the pancreas (? anatomical). Cholecystenterostomy (for hæmatemesis !) was done.

Whilst awaiting developments before operation for supposed duodenal ulcer a gentleman became jaundiced and cancer of the pancreas was discovered at operation.

Some years ago a patient was sent to the radiographer for X-ray diagnosis of possible ulcer. Whilst being X-rayed she was taken acutely ill with symptoms suggesting *perforation.* An immediate operation was done, *acute hemorrhagic pancreatitis* found, and I possess an X-ray photograph of this rare condition.

7. **Vomiting** is really of no true diagnostic value in the absence of pyloric obstruction. Nor is relief from pain after vomiting, for this relief occurs with appendix dyspepsia. It is a viscero-central reflex and may be initiated by central impulses ; for the persistent vomiting of young women, when pyloric obstruction is absent, is usually hysterical. It may be serious. In 1909 a patient died suddenly at the Bristol Royal Infirmary during an attack of vomiting.

A nurse was thought to have a possible ulcer and to require operation. In course of examination a retroverted uterus was detected by rectal examination. Its replacement cured her vomiting.

An experienced physician treated a patient for hysterical flatulence; but finding he could not cure the patient asked me to explore, as he did not wish to overlook anything. Duodenal ulcer was found.

8. **Test Meals.**—Much energy has been expended on test meals, but the results have not met expectations. Of 1,200 cases, 400 of which came to operation, the chemical and microscopical examination of the gastric contents proved of little value (W. J. Mayo[26]). S. Fenwick gives the causation of hypersecretion roughly in these proportions: ulcer 4, appendix 2, gall-stones 1,[27] and states that the symptoms of chronic duodenal ulcer occur in all forms of hypersecretion and in proportion to the excessive acidity. W. J. Mayo says that 88 per cent. of cases of hypersecretion have lesions in the digestive organs, and 12 per cent. of the cases are due to the appendix, the removal of which will generally effect a cure.[28] Our President, quoting Friedenwald, indicated that in duodenal ulcer the acidity is normal in half the cases, and of the remainder it is excessive or diminished in the proportion of 2 to 1.[29]

Mr. Jackman, Surgical Registrar at the Bristol Royal Infirmary, has tabulated the test meals of 1922, 37 tests on 30 cases, the paucity showing that the test is not in much favour now.

TEST MEALS AT THE BRISTOL ROYAL INFIRMARY DURING 1922.

| | *Acidity.* | | | |
|---|---|---|---|---|
| | + | N | – | |
| Duodenal ulcer found | — | 1 | — | |
| Gastric ulcer ,, | — | — | 1 | |
| Gall-stones ,, | — | — | 1 | |
| Cancer ,, | — | — | 1 | |
| Cholecystitis ,, | — | — | 1 | |
| Gastritis (? functional) | — | — | 1 | |
| Various (No Op., No P.-M.) | 6 | 3 | 15 | (Total 30) |

*Proportion of Free HCl to Total Acidity*
*(Normal about one-third).*

| | Over one-third. | Under one-third. |
|---|---|---|
| Duodenal Ulcer .. | 2 (1 Op.) | 0 |
| Gastric Ulcer .. | 3 (1 Op.) | 2 |

(On clinical diagnosis, except 2 operations.)

The newer and fractional methods of Rehfuss is said by Moynihan to be most informative. By this method some 20 per cent. of gastric ulcers show hyperchlorhydria against 70 per cent. of duodenal ulcers.

At the British Medical Association in 1920 it was proposed that the term "hyperacidity" should be abolished; and seeing that the mere suggestion of hunger has caused a great increase of HCl and motility in a hypnotised patient, it is obvious that central impressions play a great part in digestive secretion, and a patient may be literally "too tired to eat."

Cannon records an instructive case of a refined and sensitive woman whose test meal was completely upset by turbulent anxiety the previous night owing to her husband's drunken escapades.[30]

9. **Hunger Pain.**—The experiments of Washburn prove conclusively that hunger pain is due to contractions of the alimentary canal, the contractions rather preceding the sensations in point of time.[31] If food be eaten in hunger the stomach discharges its contents more rapidly (Haudek and Stigler), and the contractions continue after the vagus supply to the stomach has been destroyed (Carlson[32]). Hunger pain is met with in all forms of hypersecretion, and is often pronounced in biliary hypersecretion.

Some years ago a patient was sent to me with typical symptoms of duodenal ulcer—severe pain, relieved by food, and especially severe at night. At the operation there was only a breaking-down gland near the common duct and duodenum.

Two cases of right renal calculus have been recorded by Sherren causing hunger pain with high gastric acidity.

On the other hand, last May a patient had an operation for duodenal ulcer, but she had no hunger pain and her pain was relieved by vomiting.

*Appendix dyspepsia.*—This may be a definite entity or a trap for the unwary. Whenever there is the least tenderness in the appendix region, and particularly when there is a reference of the pain downwards towards the right lower abdomen, the appendix should be kept in mind. Hypersecretion and hyperperistalsis may be the effect of appendix trouble, and more active peristalsis than usual may be visible near the pylorus when the surgeon explores.

Braithwaite has associated a pyloric blush and enlargement of glands at the ileo-cæcal angle with this condition.[33] It must not be forgotten that, whatever the exact explanation may be, appendicitis may cause hæmatemesis and melæna.

Last year I removed the appendix of a woman with a ten years' history suggestive of ulcer, who had been on milk diet for three years.

Some cases have been mentioned previously under hyperalgesia and hæmatemesis. One patient had pain like a knife sticking into the abdomen for years after taking food ; she was drawn double with the pain for two hours at a time.

But mistakes may be made the other way. Some years ago I removed the appendix of a girl of about 13, who was operated upon by a colleague a few months later for perforated duodenal ulcer.

*Omental Bands* have been found in several cases in which peptic ulcer seemed possible, as in the following examples.

A gentleman was invalided home from South Africa with the diagnosis of duodenal ulcer, confirmed in London and by us here. No ulcer could be found, to our surprise, but on turning up a large mass of omentum a band was discovered passing round the transverse colon, the size of the little finger. Its excision relieved the symptoms and the patient was on active service in the War.

A medical man, dyspeptic for several years, had a single lesion—a string-like adhesion to the small intestine at its lower third. The bowel above was healthy, below it was like that of a marasmic child. Subsequent to operation he could eat his evening meal in comfort, which he could not do before.

*Abdominal tuberculosis* may mimic the symptoms of ulcer very closely.

Three years ago a patient was sent in for possible ulcer. Exploration revealed tuberculosis of the upper abdomen. The patient does not accept that diagnosis, and the Medical Defence Union is prepared to defend his threatened action.

A patient was admitted for "gastric or duodenal ulcer," the chief symptoms being vomiting and dyspepsia. There were present caseous glands at the splenic flexure, tubercles on the cæcum, and various adhesions.

There is dissatisfaction and there is hope. Dissatisfaction in our old beliefs; and that the recognition of peptic ulcer is so difficult, particularly on the gastric side of the pylorus; and hope that the X-ray diagnosis will help us in our difficulties in the future far more than in the past.

From a careful personal study of photographs during the last few months my hopes have been converted into a strong belief that more accurate conclusions are in view.

### REFERENCES.

[1] Carman, R. D., *Collected Papers of the Mayo Clinic*, 1920, xii. 41.
[2] Carman, R. D., *Ibid.*, p. 47.
[3] Moullin, Mansell, *Brit. M. J.*, 1902, ii. 1651.
[4] Langley, J. N., *The Autonomic Nervous System*, Part I., 1921.
[5] Head, H., "Certain Aspects of Pain," *Brit. M. J.*, 1922, i. 1.
[6] Keith, A., Cavendish Lecture, *Lancet*, 1915, ii. 372.
[7] Ligat, D., *Practitioner*, 1916, xcvii. 106; *Lancet*, 1919, i. 729.
[8] Sherren, J., *Lectures on the Surgery of the Stomach and Duodenum.*
[9] Hilton, *Rest and Pain*, 4th Edition, 1887.
[10] Mackenzie, Sir James, *Brit. M. J.*, 1921, i. 147; *Symptoms and their Interpretation*, 1909.
[11] Kappis, quoted by G. L. Labat, *Ann. Surg.*, 1921, lxxiv. 679 (reference not given).
[12] Crile and Lower, *Anoci-association*, 1914, 41.

[13] Hurst, A. F., *Goulstonian Lectures: on the Sensibility of the Alimentary Canal*, 1911.

[14] Wilkie, D. P. D., *Brit. J. of Surg.*, 1921, ix. 213.

[15] Duke, W. W., Quoted in *Brit. M. J.*, 1921, ii. 572.

[16] Muller, G. P., and Ravdin, I. S., *Ann. Surg.*, 1921, lxxiv. 223.

[17] Cannon, W. B., *Bodily Changes in Pain, etc.*, 1915; *The Mechanical Factors of Digestion*, 1911.

[18] Gray, Tyrrell, *Brit. M. J.*, 1922, i. 253.

[19] Paterson, H. J., *Tr. Roy. Soc. Med., Surg. Sect.*, 1910, iii. 187.

[20] Rankin, quoted by Judd, *Ann. Surg.*, 1922, lxxv. 461.

[21] Crispin, *Ibid.*

[22] Judd, E. S., *Ann. Surg.*, 1922, lxxv. 459.

[23] Deaver, quoted by Judd, *Ibid.*, p. 460.

[24] Kelling, quoted by Judd, *Ibid.*, p. 460.

[25] Irwin, S. T., *Brit. M. J.*, 1909, i. 979.

[26] Mayo, W. J., *St. Paul Medical Journal*, 1904.

[27] Fenwick, S., *Tr. Roy. Soc. Med., Surg. Sect.*, 1910, iii. 177.

[28] Mayo, W. J., *Ibid.*, quoted by S. Fenwick, p. 181, and by H. J. Paterson, p. 187.

[29] Friedenwald, quoted by J. A. Nixon, *Bristol M.-Chir. J.*, 1912, xxx. 255.

[30] Cannon, W. B., *Bodily Changes in Pain, etc.*, 1915, p. 17.

[31] *Ibid.*, p. 255.

[32] Carlson, A. J., *Amer. J. of Physiology*, 1917 and 1919.

[33] Braithwaite, Demonstration, Association of Surgeons, Leeds, May, 1922. Referred to by Moynihan, *Brit. M. J.*, 1923, i. 224.

